# Causes of cancellations on the day of surgery at a Tertiary Teaching Hospital

**DOI:** 10.1186/s12913-016-1475-6

**Published:** 2016-07-13

**Authors:** Roland Kaddoum, Racha Fadlallah, Eveline Hitti, Fadi EL-Jardali, Ghada El Eid

**Affiliations:** Deparment of Operating Room, American University of Beirut Medical Center, Beirut, Lebanon; Department of Health Management and Policy, Faculty of Health Sciences, American University of Beirut, Beirut, Lebanon; Department of Emergency Medicine, American University of Beirut Medical Center, Beirut, Lebanon; Department of the Chief Medical Officer, American University of Beirut Medical Center, Beirut, Lebanon

**Keywords:** Operating rooms, Day of surgery, Causes of cancellations, Avoidable and unavoidable causes

## Abstract

**Background:**

Cancellation of elective scheduled operations on the day of surgery leads to an inefficient use of operating room (OR) time and a waste of resources. It also causes inconvenience for patients and families. Moreover, day of surgery (DOS) cancellation creates logistic and financial burden associated with extended hospital stay and repetitions of pre-operative preparations as well as opportunity costs of lost time and missed income. The objective of this study is to establish the rate of elective surgical cases cancellations on the day of surgery and the reasons for these cancellations stratified by avoidable versus unavoidable within a tertiary care teaching hospital in Beirut, Lebanon as well as recommend appropriate solutions.

**Method:**

This is a prospective audit of the operation theatre list over a period of eight months (January 1, 2013-August 30, 2013). All patients scheduled to undergo elective surgeries at the hospital from January-August 2013 were included. An assigned OR staff recorded the cancelled cases in real time. The assigned staff confirmed the cancellation reason and added additional explanation if necessary by calling patients or through direct inquiry of clerical and/or clinical staff the following day. A Pareto chart was constructed to prioritize the reasons that accounted for 80 % of the avoidable surgical cancellations.

**Results:**

For the given study period, 5929 elective surgeries were performed, of which 261 cases (4.4 %) were cancelled on the day of surgery. 187 cases (or 71.6 %) were judged as potentially avoidable cancellations versus 74 (28.4 %) that were judged as unavoidable. Of the 187 potentially avoidable cancellations, lack of financial clearance, incomplete medical evaluation, patient not showing up for surgery, and OR behind schedule accounted for almost 80 % of the causes.

**Conclusion:**

This study showed that the majority of cancellations were deemed avoidable and hospital related. A day of surgery cancellation rate less than 2 % is attainable. Determining the major avoidable contributors to DOS cancellations is an essential first step to developing appropriate interventions to improve operating theater efficiency. Recommended interventions were presented accordingly.

## Background

Cancellation of elective scheduled operations on the day of surgery leads to an inefficient use of operating room (OR) time and a waste of resources. It also causes inconvenience for patients and families [1]. Moreover, day of surgery (DOS) cancellation creates logistic and financial burden associated with extended hospital stay and repetitions of pre-operative preparations as well as opportunity costs of lost time and missed income [2–5].

DOS cancellations are a world-wide problem with reported rates ranging from 1.96 to 24 % [6, 7]. There are numerous reasons for cancellations of elective surgical cases and these vary from one hospital to another [8]. The range of reasons given include inadequate pre-op assessment and preparation [9], patient-related factors [7], lack of operating room time [10], unavailability of hospital beds [6], prediction bias [11], surgeon-related issues [12] and emergency surgery disrupting the elective list [13]. These are traditionally divided into potentially avoidable cancellations (e.g. cancellation due to inadequate preoperative evaluation, scheduling errors, and equipment shortages) and unavoidable cancellations (e.g. changes in patients’ medical status, and emergency case superseding the elective schedule) [10]. A significant percentage of day of surgery cancellations are rendered avoidable [6, 9].

Many setting-specific factors contribute to the variability; from differences in reimbursement systems to patient and staff characteristics as well as differences in work ethic and culture. This is the first study to look at DOS cancellations in the Lebanese setting.

The objectives of this prospective study were to establish the rate of elective surgical cases cancellations on the day of surgery and the reasons for these cancellations within a tertiary care hospital in Lebanon as well as to recommend appropriate solutions to improve the operating theatre efficiency. Additional measures include avoidable versus unavoidable day of surgery cancellations.

## Methods

### Study design and setting

The American University of Beirut Medical Center (AUBMC) is a non-profit tertiary care teaching hospital in Lebanon with a bed capacity of 384. The Operating Room (OR) has 11 suites distributed among eleven specialties: general surgery, cardiac surgery, orthopedics, ophthalmology, neurology, gynecology, urology, vascular surgery, pediatric, plastic, and Otolaryngology. Different specialties are allotted different rooms on specific days of the week with no room reserved exclusively for emergency and life-saving cases.

The typical start time for elective surgery is set at 8.00 AM. By definition, elective surgeries are those that are planned for in advance and scheduled any time before 2.30 PM of the day preceding the surgery date. The addition of late elective cases or shuffling of cases after that time needs the approval of the OR medical director who oversees the entire surgery list.

We defined DOS cancellation as an operation that was scheduled on the final OR list for that day (generated at 2.30 PM on the previous day) and was subsequently cancelled on the day of surgery. For the purpose of this study, we limited cancellation analysis to only elective surgeries that were cancelled on the day of intended surgery. Emergency, life saving and minor ambulatory surgery done outside the OR unit were excluded.

To establish the reasons for DOS cancellations of elective cases, we conducted a prospective audit of the operation theatre list over a period of eight months (January 1, 2013-August 30, 2013). All patients scheduled to undergo elective surgeries were prospectively enrolled into the study. Data on surgeries scheduled for weekdays, excluding public holidays, were obtained on a daily basis from the operation theatre list for that day, typically generated at 2.30 pm the previous day. The list is electronically updated at the end of the day by the OR staff to accommodate for any changes that may have occurred throughout the day. The list provides details on patient ID number, surgeon name, intended procedure, operating theater room, and estimated duration of each surgery.

For the purpose of this study, data was collected on the following variables: the number of scheduled elective surgeries, the number of cancelled elective surgeries, the reasons for cancellations, and the type of admission. Reasons for cancellation were placed into one of the following six predefined categories: (1) Work-up/medical condition change, (2) patient related, (3) financial, (4) Facility/Resources, (5) Bed availability, and (6) surgeon related.

The reasons for cancellation were provided by either the surgeon or the resident and prospectively recorded into the computerized database. The assigned OR staff confirmed the cancellation reason and added additional explanation if necessary by calling patients or through direct inquiry of clerical and clinical staff the following day. Specifically nineteen patients were called to validate reasons related to pre-operative instruction and patients changing their minds last minute on the DOS. For cancellations where the recorded reason was inconsistent with that reported by the surgeon/resident, the assigned theatre staff informed the OR medical director who, in turn, initiated an investigation to arrive at the correct reason. A Quality Improvement (QI) team was assigned comprising the OR medical director, an expert in lean six sigma and the OR nurse manager to oversee the whole project. Consensus was reached to avoid assigning a single cancellation to more than one category. While this may be a bit challenging given the complex nature of operating theatres, the OR team attempted to address this by agreeing to assign the root cause to a cancellation.

Cancellations were further classified as potentially avoidable and non-avoidable. Avoidable cancellations were defined as those cancellations that could have been avoided had there been adequate review of patients’ medical records or communication by hospital personnel before the day of surgery. We also divided the reasons for cancellations into hospital versus patient related causes. In thirteen cases where we were uncertain as to which category to assign the reason (avoidable versus non-avoidable, hospital versus patient), the QI team discussed the cases and reached consensus on the final decision.

To prioritize future improvement efforts, we constructed a Pareto chart and identified the reasons that account for 80 % of the *avoidable* surgical cancellations. Descriptive and basic analytical statistics were used to summarize the data.

The study was deemed exempt from human subject research by the Institutional Review Board of the American University of Beirut. It was determined that, since this was a quality improvement project and information collected did not include personal identifiers, individual consents were not required.

## Results

During the study period, 5,929 elective surgeries were performed, of which 261 were cancelled on the day of surgery. This yielded a mean cancellation rate of 4.4 % per month (4.4 % ± 1.4 SD). The most common category for DOS cancellation was work-up/medical condition which accounted for 85 cancelled cases (or 32.6 % of all elective surgical cancellations). This was followed by resource/facility (*n* = 52 or 20 %), admission (*n* = 50 or 19.2 %), patient (*n* = 46 or 17.6 %), bed (*n* = 21 or 8 %), and surgeon (*n* = 7 or 2.7 %). The most common single cancellation reason was “no financial clearance” (*n* = 43) followed by “incomplete medical evaluation“(*n* = 34) and “change in medical status” (*n* = 31), respectively (see Table 1).

**Table 1:** Frequency and percentage of day of surgery cancellations by categorical reasons

Patient (*n* = 46 or 17.6 %) • Patient did not show up for surgery (*n* = 29) • Patient changed mind about surgery (*n* = 7) • Patient had a panic attack (*n* = 1) • Patient refused surgery (*n* = 4) • Patient’s family refused surgery (*n* = 2) • Patient left against medical advice (*n* = 1) • Patient Postponed (*n* = 2)	Resource/Facility (*n* = 52 or 19.9 %) • OR behind schedule (*n* = 23) • Equipment broken or not available (*n* = 6) • Implant(s) not available (*n* = 1) • Emergency/life-saving case prioritized (*n* = 3) • Scheduling error (*n* = 5) • Staff miscommunication (*n* = 13) • Pre-operative instruction not communicated properly to patient (*n* = 1)
Workup/Medical condition (*n* = 85 or 32.6 %) • Change in medical status (*n* = 31) • Incomplete Surgical-work up (*n* = 4) • Incomplete Medical evaluation (*n* = 34) • Abnormal test (*n* = 5) • Change in treatment plan (*n* = 6) • Difficult unobtainable IV access (*n* = 1) • Pre-operative instructions not followed properly (*n* = 4)	Surgeon (*n* = 7 or 2.7 %) • Surgeon not available (*n* = 4) • Surgeon sick (*n* = 1) • Surgeon was operating in another hospital (*n* = 1) • Surgeon was travelling (*n* = 1)
Admission (*n* = 50 or 19.2 %) • No Financial Clearance (*n* = 43) • Papers not ready (*n* = 7)	Bed (*n* = 21 or 8 %) • No private bed available (*n* = 3) • No government-contracted bed available (*n* = 17) • No cardiac surgery unit bed (*n* = 1)

Total number of elective surgical cancellations (*n* = 261)

**Fig. 1:**
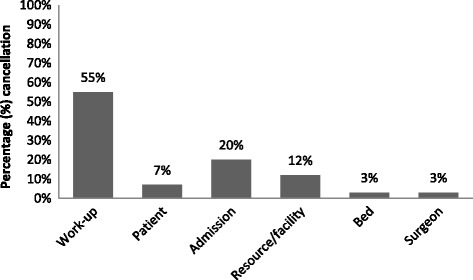
Percentage of first case cancellations by reason

Of the 261 total cancelled elective surgeries, 40 (or 15 %) were scheduled as first cases. Of these, work-up/medical condition changes accounted for the highest cancellations (Fig. 1).

**Fig. 2:**
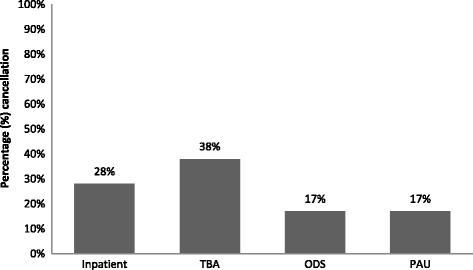
Percentage of elective surgical cases cancellations by admission type. Abbreviations: PAU: Pre-operative Assessment Unit; ODS: One Day Surgery Unit; TBA: To Be Admitted (main admission unit)

Stratification of cancellations by admission type indicated that inpatients and to-be-admitted patients (TBA) accounted for 172 elective cancellations (66 %) relative to patients admitted via the pre-operative assessment unit (PAU) or one-day surgery (ODS) patients (Fig. 2).

One hundred eighty-seven DOS cancellations (71.6 %) were judged as potentially avoidable while 74 cases (28.4 %) were judged as unavoidable (see Table 2). Of the medical-related cancellations (*n* = 85), incomplete surgical-work up (*n* = 4) and incomplete medical evaluation (*n* = 34) were judged as potentially avoidable. Of the patient-related cancellations (*n* = 46), patient did not show up for surgery (*n* = 29) was judged as potentially avoidable. As for the cancellations under resource/facility (*n* = 52), the following reasons were judged as avoidable: equipment broken or not available (*n* = 6), implant not available (*n* = 1), scheduling error (*n* = 5), staff miscommunication (*n* = 13), and pre-operative instruction not communicated properly to patient (*n* = 1). All other cancellations under “bed”, “admission” and “surgeon” category respectively, were judged as potentially avoidable (see Table 2).

**Table 2:** Day of surgery cancellations stratified by avoidable versus unavoidable causes

	Avoidable (*n* = 187or 71.6 %)	Unavoidable (*n* = 74or 28.4 %)
Hospital Related (*n* = 174 or 66.7 %)	▪ Incomplete surgical-work up (*n* = 4) ▪ Incomplete medical evaluation (*n* = 34) ▪ Staff miscommunication (*n* = 13) ▪ No financial clearance (*n* = 43) ▪ Admission papers not ready (*n* = 7) ▪ OR behind schedule (*n* = 23) ▪ Scheduling error (*n* = 5) ▪ Implants not available (*n* = 1) ▪ No bed available (*n* = 21) ▪ Equipment unavailable (*n* = 6) ▪ Patient not instructed appropriately to stop drinking water (*n* = 1)	▪ Change in treatment plan (*n* = 6) ▪ Emergency/life-saving case that occupied the OR (*n* = 3) ▪ Surgeon not available (*n* = 4) ▪ Surgeon was operating in another hospital (*n* = 1) ▪ Surgeon was travelling (*n* = 1) ▪ Surgeon sick (*n* = 1)
Patient Related (*n* = 87 or 33.3 %)	▪ Patient did not show up for surgery (*n* = 29)	▪ Change in medical status (*n* = 31) ▪ Abnormal test (*n* = 5) ▪ Difficult unobtainable IV access (*n* = 1) ▪ Patient family refused surgery (*n* = 2) ▪ Patient postponed (*n* = 2) ▪ Patient left against medical advice (*n* = 1) ▪ Patient changed mind about surgery while in hospital (*n* = 7) ▪ Patient had a panic attack (*n* = 1) ▪ Patient refused surgery (*n* = 4) ▪ Preoperative instructions not followed properly (*n* = 4)

*n* = 261

As shown from Table 2, the greatest numbers of DOS cancellations were deemed *avoidable* and *hospital*-related (upper left quadrant, Table 2). Of the 158 cases in this category, the most common reason for cancellation was no financial clearance (*n* = 43), i.e. where the patient presented on the day of surgery with no insurance approval, was unable to pay cash, or the admission staff had not discussed the finances adequately ahead of time. These were judged as avoidable since, at the time the study was conducted, there was no system in place to check the financial status of the patient prior to the surgery date. Patient incomplete medical evaluations represented the second most common event under this category accounting for a total of 34 cancelled cases (examples include no cardiac clearance, no pulmonary clearance, no endocrinology clearance, patient needs MRI, etc.). These cancellations were mainly due to the pre-operative assessment commencing on the evening preceding surgery, with some of the assessments shifting to the day of surgery. Miscommunications among staff members or between the physicians and patients accounted for another 13 cancelled cases. These included patients not being informed of the correct surgery time or the surgery being transferred to another unit but still appearing on the schedule because the OR staff was not informed. Bed availability represented 21 out of 158 cancellations in this subset; of these, 17 were cancelled due to limited government-contracted beds and 3 due to the unavailability of private beds with only a single cancellation resulting from the cardiac surgery unit being full. Cancellation or postponement of surgery because the OR was behind schedule accounted for another 23 cases. These were mainly due to overbooking of the surgery list, thus resulting in long schedules and subsequently more cases ending up being cancelled towards the bottom of the list. A second reason related to the surgeons’ inaccurate estimation of surgery durations. While it may be argued that the latter could not be predicted accurately due to unexpected complications that may arise, this was not always true, and in our case, surgeons could have factored such “uncertainty” when informing the OR staff of the estimated surgery durations.

The only *avoidable patient-related* cancellations were patients not showing up for surgery. These were judged as potentially avoidable since at the time the study was conducted there was no any standardized process or systematic approach to contact patients a day prior to their surgery dates to confirm their attendance.

Of the *unavoidable* cancellations, 16 cases (21.6 %) were hospital-related and 58 cases (78.4 %) were patient-related. The majority of the unavoidable hospital-related cancellations were due to the unavailability of surgeons or surgeons’ decisions to change treatment plan. Of the 58 unavoidable patient-related cancellations (lower right quadrant, Table 2), 31 were due to changes in patient medical status, e.g. the patient presented to surgery with an upper respiratory or urinary tract infection or a new cardiac dysrhythmia. Abnormal lab values (e.g. unexpected high Creatinine, high white count) on the day of presentation resulted in 5 cancellations.

**Fig. 3:**
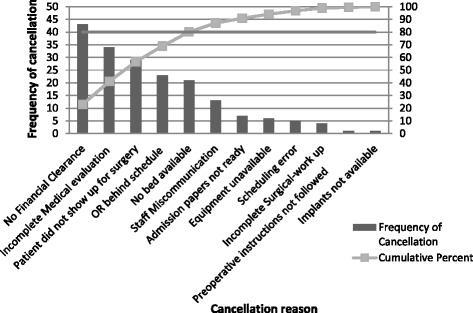
Pareto chart of Avoidable surgical cases cancellations by reason

To prioritize the reasons contributing to the highest *avoidable* cancellations, we constructed a Pareto Chart. The Pareto Chart showed that no financial clearance, incomplete medical evaluation, patient not showing up for surgery, OR behind schedule and no beds available accounted for 80 % of all *avoidable* same-day cancellations of elective surgeries (Fig. 3). Thus, any improvement to reduce day of surgery cancellation should target the aforementioned five reasons.

## Discussion

DOS cancellation is a world-wide problem with reported incidences ranging from less than 1 % to over 23 % [9, 14]. The percentage of DOS cancellations at our hospital was found to be 4.4 %. This is comparable to another study in a Jordanian hospital where the rate of DOS cancellation was reported to be 3.6 % [15]. While no consensus has been reached on the acceptable cancellation rate, less than 5 % is generally recommended [16]; however, DOS cancellations of less than 2 % have been reported [17].

Based on our findings, work-up/medical related issue (32.6 %) was the most common category for DOS cancellations. This was comparable to the findings from other studies conducted in the US, Spain, Taiwan, and Jordan [9, 15, 18]. Resource/facility was the second most common category for DOS cancellations (19.9 %) followed by admission (19.2 %), patient-related reasons (17.6 %), bed availability (8 %), and surgeon-related reasons (2.7 %). Similar results have been reported elsewhere. For example, in a Jordanian tertiary hospital, medical reasons, patient-related reasons (non-attendance) and administrative (unavailability of hospital beds) reasons accounted for 38.2, 31.4 and 30.4 % of all cancelled elective operations, respectively [15]. A teaching hospital in Hong Kong reported the following causes of elective DOS cancellations: facility (73 %), work-up (17 %), patient (10 %), and surgeon (1 %) [19]. In a study conducted in Spain, the authors reported the following reasons for DOS cancellations: medical reason (50 %), administrative/logistic reasons (24.7 %), and patient-related causes (23.1 %) [9]. In the US setting, patient (44.4 %), work-up (20 %), capacity (11.1 %), unavailability of specialized personnel (11.1 %) and no real cancellation, e.g. scheduling errors (11.1 %) comprised the causes of cancellations on the day of surgery at a major university hospital [2].

Most of the DOS cancellations of elective cases at our hospital were deemed avoidable and hospital-related. The Pareto chart highlighted the following reasons as contributing to the majority of the avoidable DOS cancellations: no financial clearance, incomplete medical evaluation, patient not showing up for surgery, OR behind schedule and no beds available. This has implications for practice particularly with respect to prioritizing interventions to reduce DOS cancellations. Below, we briefly discuss the causes of each of these cancellations and where applicable, offer potential solutions.

i. Financial clearance
  The most common potentially *avoidable* cancellations at our medical center were cases that were cancelled due to no financial clearance i.e., the patient presented to the day of surgery with no insurance approval, was unable to pay, or the admission staff had not completed the financial clearance of the patient. This was not a surprise given the absence of universal healthcare coverage in Lebanon. Consequently, almost half of the population is not covered by any formal insurance [20].
  Further analysis revealed that there are no policies in place requiring financial clearance to be obtained at a specific time prior to surgery. Consequently, the patients remain scheduled in the final OR list of elective surgeries (distributed at 2.30 pm of the day preceding the surgery date) regardless of whether they have obtained financial clearance. To address this issue, the hospital is working on developing a software system that makes the financial status of patients visible to both operating room and admission office staff; thus allowing them to remove the case from the schedule if the financial status of the patient is not confirmed by 6 pm of the day preceding the scheduled surgery.
ii. Incomplete medical evaluation
  The majority of patients in our study was either admitted one day prior to the surgery or was existing inpatients. In spite of this, incomplete surgical or medical pre-operative assessments accounted for the second most common causes of *avoidable* cancellations. This is attributed to the absence of explicit policies at our hospital that outline the expected timelines for completion of pre-operative assessments, with some of the assessments left to the day of the surgery. Further investigations revealed that only 34 % of our patients were admitted through the pre-operative assessment unit (PAU) (data not shown), with the majority of the latter failing to present at least 3-10 days prior to their surgeries for medical clearance as per the PAU policy.
  Substantial literature exists on the benefits of establishing a pre-operative assessment unit/clinic where patients complete their pre-operative assessments and obtain medical clearance few days before their surgery date. The evidence shows that PAU not only significantly decreases cancellations and delays of elective cases [21–23], but it also enhances patient satisfaction [24], significantly reduces unnecessary pre-operative testing and consultation [25] as well as cuts down on the length of hospital stay due to decrease in pre-operative admissions and post-operative morbidities and complications [26–28]. Work-up related reasons alone were also responsible for over 50 % of first case cancellations (Fig. 2).
iii. Patient did not show up for surgery
  Patients failing to present is a particularly challenging problem. It may be due to the patients’ last minute doubts and fears or patients forgetting about the date of their surgeries. The latter is plausible since at our hospital, there is no standardized procedure to call patients and remind them of their appointments. Rather, it is done on an ad-hoc basis and at a time that might not be convenient to patients. Reminder phone calls by an assigned personnel have been shown to improve patient compliance with appointment keeping [29] and would be a reasonable intervention in our setting where most patients have mobile phones.
iv. OR behind schedule
  Lack of OR time due to over-running of previous surgeries or emergency surgeries accounted for 12.2 % of the avoidable causes of DOS cancellations at our hospital. In Schofield et al. 18.7 % of DOS cancellations were due to over-run of previous surgery [6]. Block schedule is one intervention that has been shown to reduce this problem by improving OR scheduling [17].
  Another intervention suggested by Litvak E [30] is managing variability to optimize patient flow. Some patient flow variability is natural, such as the flow of patients admitted to a hospital unit through the emergency department. Other patient flow variability is artificial such as the flow of elective admission which, though neither random nor completely predictable, can be optimized with better management and scheduling improvement [31]. A study by Pandit & Carey [32] that looked at scheduling issues related to surgeon estimates found that though surgical guesstimates were very accurate in forecasting the actual duration of the list (*r*^*2*^ = 0.61; p < 0.001), but they were deficient at reserving the list to within its scheduled duration with 50 % of surgeons over-running their scheduled times. Use of individual surgeon’s historical estimates is one way to improve scheduling problems related to predictable over-runs and subsequent cancellations.
v. No beds available
  The majority of cancellations under this category were due to the non-availability of government-contracted beds. The latter has its root in politics and as a result, creating a mismatch in the patients to beds ratio with only 24 beds (2 beds per room) available to accommodate for the relatively higher patient loads. While this does not affect inpatients, it has implications on the to-be admitted (TBA) and PAU patients who are typically admitted after surgery. The literature recommends strengthening the discharge process and communicating the estimated date of discharge to patients and involved personnel ahead of time [7, 33].

### Limitations

A key weakness of this study relates to the inherent bias in categorizing cases where there was some ambiguity or where multiple factors may have contributed to the cancellation. We attempted to minimize this by relying on a team consensus approach to make the final assessment in these cases rather than relying on one individual. Such cases were however few. Finally, the study was limited to one hospital in the Lebanese health system and may not be generalizable to other hospitals and other healthcare systems.

## Conclusions

DOS cancellation rate in our study was found to be 4.4 %. Over 70 % of the elective surgical cancellations had avoidable causes with lack of financial clearance, incomplete pre-operative assessments, patient not showing up for surgery and OR behind schedule being the most common avoidable causes of cancellations. Determining what the major avoidable contributors to DOS cancellations is an essential first step to developing appropriate interventions to improve operating theater efficiency.

## Abbreviations

AUBMC, American University of Beirut Medical Center; DOS, day of surgery; ODS, one-day surgery; OR, operating room; PAU, pre-operative assessment unit; QI, quality improvement; TBA, to-be-admitted
